# Krüppel-like factor 4 promotes human osteosarcoma growth and metastasis via regulating CRYAB expression

**DOI:** 10.18632/oncotarget.8824

**Published:** 2016-04-18

**Authors:** Lu Zhang, Li Zhang, Xin Xia, Shengwei He, Hongtao He, Wenzhi Zhao

**Affiliations:** ^1^ Department of Orthopedics, The Second Affiliated Hospital of Dalian Medical University, Dalian 116027, China; ^2^ Laboratory of Pathogenic Biology, College of Basic Medical Science of Dalian Medical University, Dalian 116027, China; ^3^ Department of Orthopedics, The Second Affiliated Hospital of Dalian Medical University, Dalian, Liaoning 116027, China

**Keywords:** KLF4, osteosarcoma, migration, CRYAB, proliferation

## Abstract

Krüppel-like factor 4 (KLF4), a zinc-finger transcription factor, is an essential regulator in many cellular processes, including differentiation, proliferation, inflammation, pluripotency, and apoptosis. Along with these roles in normal cells and tissues, KLF4 has been reported as a tumor suppressor or an oncogene in many cancers. However, the role of KLF4 in osteosarcoma is largely unknown. Here we found the expression of KLF4 was significantly increased in human osteosarcoma tissues compared with the normal tissues. Elevated KLF4 promoted human osteosarcoma cell proliferation and metastasis. Subsequently, mechanistic studies revealed KLF4 specifically bound the promoter of CRYAB and upregulated CRYAB expression in human osteosarcoma cells. Moreover, we found that KLF4 enhanced osteosarcoma cell proliferation and migration via upregulating CRYAB. Therefore, our studies suggested KLF4 may be a potential target for human osteosarcoma therapy.

## INTRODUCTION

Osteosarcoma (OS) is the most common type of malignant bone cancer that usually occurs in children and young adults [18, 19]. Although, remarkable advances in the combined used of chemotherapy, radiotherapy and surgical ablation of the primary tumor, the 5-year survival rate of osteosarcoma patients is still not more than 30% due to its metastasis and recurrence [11]. Thus, it is very important to uncover the molecular mechanisms by which OS initiates, proliferation, metastasis, and recurrence to develop effective therapeutic strategies for treatment of this disease.

Krüppel-like factor 4 (KLF4), also known as gut-enriched Krüppel-like factor, is a zinc-finger transcription factor which regulates various biological processes, including cell cycle progression, differentiation, proliferation, inflammation, apoptosis and stem cell renewal [1, 2, 9, 12, 21, 29]. As a transcription factor, KLF4 has been reported to active or repress a lot of genes that involved in these biological processes [20]. Recent studies have described an ambivalent role for KLF4 in cancer progression [24]. Many evidence have shown that KLF4 expression is significantly reduce in a large number of human cancers including gastrointestinal, oesophageal, lung, pancreatic, colorectal, prostate, B-lymphocyte, and bladder cancers. Consistent with its expression in these tumors, KLF4 plays an active role as a tumor suppressor [3, 10, 15–17, 25, 26]. In addition, in some other cancers such as breast and oral squamous cell carcinoma, KLF4 was found to be increase and act as an oncogene [6, 8, 23, 28]. Therefore, KLF4 may function as either tumor suppressor or oncogene depending on the tissue type. However, in human osteoasarcom, its role is largely unkonwn.

In this study, we reported that KLF4 facilitated human osteosarcom cells growth and migration *in vitro* or *in vivo*. Whereafter, mechanistic studies indicated that KLF4 specifically bound the promoter of CRYAB and increased CRYAB expression in human osteosarcoma cells. Moreover, we found that KLF4 enhanced osteosarcoma cell proliferation and migration through upregulating CRYAB. Thus, our data suggest that KLF4 may be an oncoprotein in human osteosarcoma and could be a valuable target for developing treatments for patients with osteosarcoma.

## RESULTS

### KLF4 increases human osteosarcoma cell proliferation and tumorigenesis

**Figure 1:**
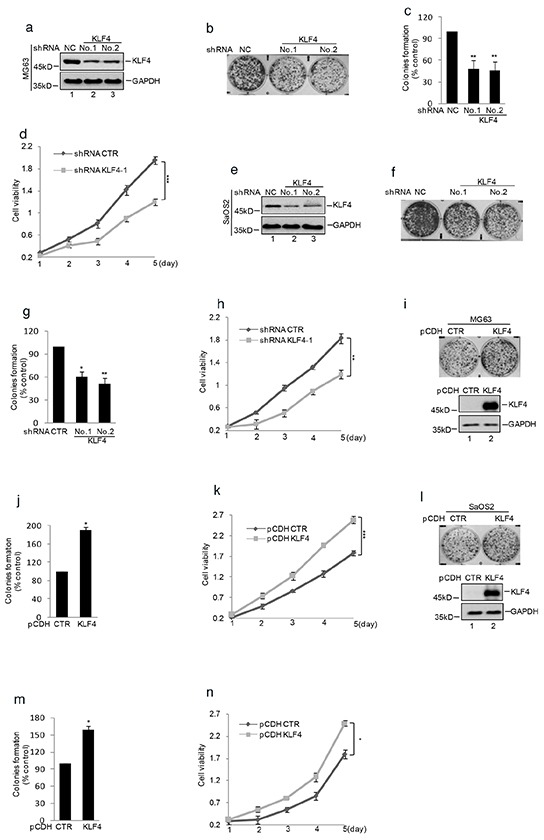
KLF4 enhanced human osteosarcoma cells clonogenicity and growth shRNA mediated silencing of KLF4 expression in human osteosarcoma cell lines. **a.** and **e.** Western blot detected the expression of KLF4 in MG63 and SaOS2 cells. GAPDH was detected as loading control. **b–c.** and **f–g.** Colony formation assay was used to measure the clonogenicity of MG63 and SaOS2 cells with or without knockdown KLF4. The numbers of control cells were set as 100%.(n=3, mean ± SD, t-test, n**P<0.01 vs. shRNA CTR). **d.** and **h.** Cells with or without knockdown KLF4 were cultured for the days as indicated and cell growth was evaluated by CKK8 assay. Results are representative of three independent experiments. *P<0.05, **p<0.01 and ***p<0.001 vs Ctr. **i–j.** and **l–m.** Colony formation assay was used to measure the clonogenicity of MG63 and SaOS2 cells with or without overexpression of KLF4. The numbers of control cells were set as 100% (n=3, mean ± SD, t-test, **P<0.01 vs. CTR). Western blot was used to detect the expression level of KLF4. **k.** and **n.** CKK8 assay was used to measure the cell viability of MG63 and SaOS2 with or without overexpression of KLF4.

To investigate the role of KLF4 in human osteosarcoma, we decreased KLF4 expression using shRNA in human osteosarcoma cells MG63 and SaOS2. As shown in Figure 1a and 1e, the expression level of KLF4 was efficiently down-regulated in comparison with the control cells. Then, we determined the effect of KLF4 on cell proliferation and tumorigenesis and found that inhibition of KLF4 expression suppressed cell growth and colony formation in human osteosarcoma cells (Figure 1b–1d and Figure 1f–1h). Whereas, overexpression of KLF4 in MG63 and SaOS2 cells significantly increased cell growth and colony formation compared with the control cell (Figure 1i–1n).

**Figure 2:**
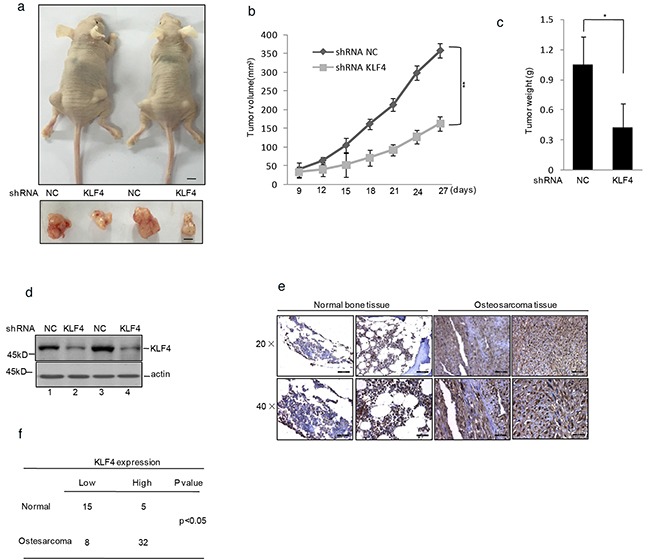
KLF4 promoted human osteosarcoma cells tumorigenicity in a nude mice model **a.** shRNA KLF4 or shRNA NC infected MG63 cells were injected subcutaneously into either side of the posterior flank of the same nude mouse (n =6 per group). The tumor size is shown in Figure 2a. The black bar represents 1 cm. **b.** The tumor growth curve is displayed in Figure 2b. **c.** The final tumor weight is shown in Figure 2c. **d.** Western blot decided the efficiency of KLF4 in the tumors shown in Figure 2a. **e–f.** KLF4 protein level in osteosarcoma and normal bone tissues was detected by immunohistochemical staining.

The effect of KLF4 on tumor growth was further identified in nude mice with human osteosarcoma xenografts. Human osteosarcoma cells MG63 with or without stably knockdown KLF4 were injected subcutaneously into the groin of nude mice. As shown in Figure 2a–2c, the tumor volume and weight of mice with the injection of KLF4 stably knockdown cells were much smaller and lighter compared with control group. Afterwards, the knockdown efficiency of KLF4 was verified by western blot (Figure 2d).

To confirm that, we used immunohistochemistry to determine the expression of KLF4 in human osteosarcoma tissues and the healthy bone tissues. As shown in Figure 2e and 2f, the expression of KLF4 was significantly increased in osteosarcoma tissues compared with the normal bone tissues.

Taken together, these data suggested KLF4 is a potential oncoprotein in human osteosarcoma.

### KLF4 promotes human osteosarcoma cell migration

**Figure 3:**
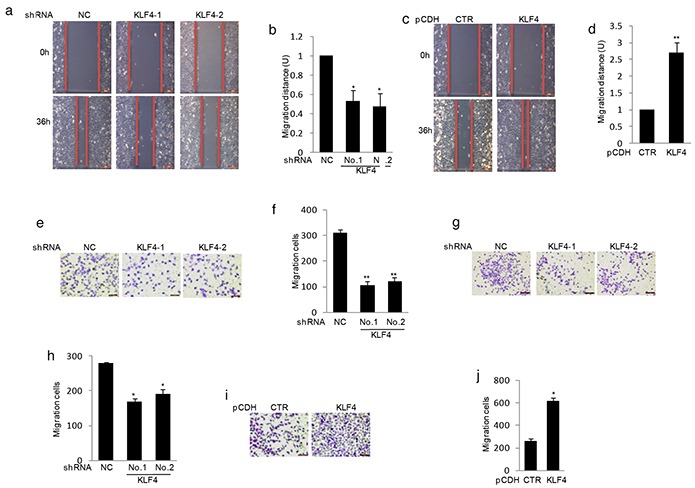
KLF4 elevated human osteosarcoma cells migration **a–d.** KLF4 were overexpressed or knockdown in MG63 cells using lentivirus vectors. The effects of KLF4 on cell migration were examined by wound-healing assay. Bar graph quantification of migration distance. U, units. Results are representative of three independent experiments. *P<0.05, **p<0.01 and ***p<0.001 vs Ctr. **e–g.** Effects of KLF4 on the migration were examined by Matrigel migration assay in MG63 (e) and SaOS2 cells (g). Migrated cells were plotted as the average number of cells per field of view. Results are representative of three independent experiments. *P<0.05, **p<0.01 and ***p<0.001 vs Ctr. **i–f.** KLF4 were overexpressed in MG63 cells and its effect on cell migration were examined by Matrigel migration assay. Results are representative of three independent experiments. *P<0.05, **p<0.01 and ***p<0.001 vs Ctr.

As activation of metastasis is one key hallmark of tumor cells, we next investigated the effect of KLF4 on the process. The wound healing assay indicated that knockdown KLF4 gene dramatically inhibited cell migration (Figure 3a–3b). Conversely, overexpression of KLF4 enhanced cell migration (Figure 3c–3d). We then adopted another methodology, Matrigel migration assay, to confirm our findings. Consistent with the previous result, data from Matrigel migration assay showed that shRNA mediated KLF4 depletion markedly suppressed cell migration in MG63 and SaOS2 cells (Figure 3e–3h). Whereas, upregulation of KLF4 increased cell migration in MG63 cells (Figure 3i–3j).

### KLF4 upregulates CRYAB expression in human osteosarcoma cell

**Figure 4:**
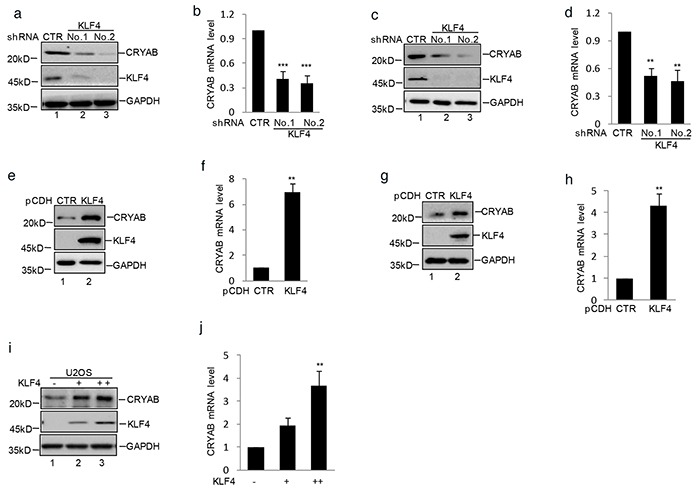
KLF4 upregulated CRYAB expression in human osteosarcoma cells **a–d.** MG63 and SaOS2 cells with or without stable knockdown KLF4 were generated. The expression level of CRYAB and KLF4 were analyzed by western blot and q-RT-PCR. Results are representative of three independent experiments. *P<0.05, **p<0.01 and ***p<0.001 vs Ctr. **e–h.** MG63 and SaOS2 cells with or without stable overexpressing KLF4 were generated using lentivirus vector. The expression level of CRYAB and KLF4 were analyzed by western blot and q-RT-PCR. **i–j.** Increasing amounts of KLF4 were transfected into U2OS cells. The expression levels of CRYAB and KLF4 were measured by western blot and q-RT-PCR.

To uncover the molecular mechanism that KLF4 involved in human osteosarcoma cell growth and migration, MG63 cells with or without stably knockdown KLF4 were subjected to cDNA microarray analysis to identify the potential associated genes. From the array data, we observed that CRYAB was dramatically down- regulated in KLF4 knockdown cells (data not shown). Real-time RT-PCR and western blot analysis showed that the mRNA and protein level of CRYAB were significantly decreased in MG63 and SaOS2 cells (Figure 4a–4d). Moreover, overexpression of KLF4 resulted in increasing CRYAB expression at both mRNA and protein level in MG63 and SaOS2 cells (Figure 4e–4h). Similarly, ectopic expression of KLF4 in U2OS cells which owned low level of KLF4, led to CRYAB elevation at both mRNA and protein level in a dose-dependent manner (Figure 4i–4j). Taken together, these data indicate that KLF4, as a transcription factor, may increase CRYAB expression in human osteosarcoma cells.

### KLF4 transactivates CRYAB gene expression by binding the CRYAB promoter region (nt -479 to -463)

**Figure 5:**
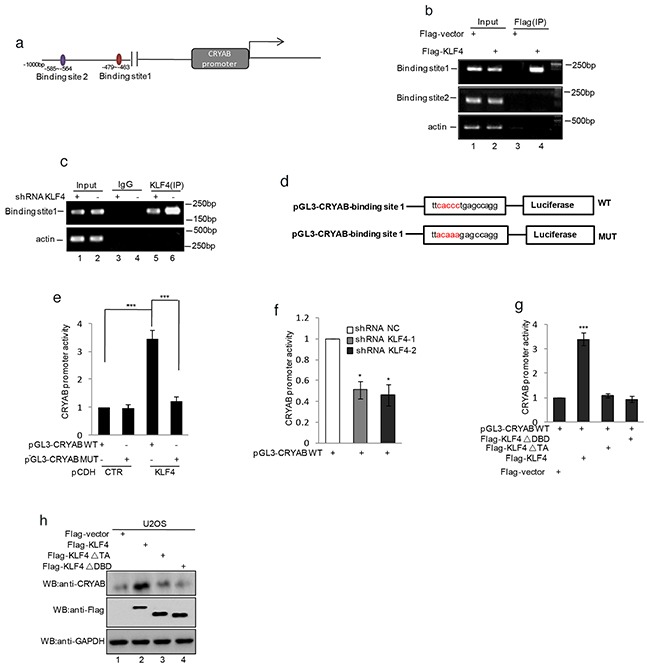
KLF4 transactivated CRYAB gene expression by binding the promoter region **a.** Schematic illustration of the putative KLF4-binding region located at 585-564 and 479-463 bp upstream of the CRYAB translational start site. **b.** ChIP analysis showed binding of KLF4 to the putative binding sites. Input and immunoprecipitate from MG63 cells with or without overexpressing KLF4 were amplified by PCR with primer pairs complementary to the CRYAB promoter. **c.** ChIP analysis showed binding of KLF4 to the putative binding sites. Input and immunoprecipitate from MG63 cells with or without knockdown KLF4 were amplified by PCR with primer pairs complementary to the CRYAB promoter. **d.** Schematic illustration of PGL3-basic-based reporter constructs used in luciferase assays to examine the transcriptional activity of the binding site 1. The red sequences indicate the mutated nucleotide residues. **e.** MG63 cells with or without overexpressing KLF4 were transfected with pGL3-CRYAB WT or pGL3-CRYABMUT. 24h after transfection, transcription activity was determined with dual-luciferase assay. **f.** MG63 cells with or without knockdown KLF4 were transfected with pGL3-CRYAB WT. 24h after transfection, transcription activity was determined with dual-luciferase assay. **g–h.** U2OS cells were transfected with pGL3-Siat7A WT as well as flag-KLF4, flag- KLF4▵TA (deletion of the transcription activity domain), or flag- KLF4▵DBD (deletion of the DNA binding domain). 24 h after transfection, transcription activity was determined with dual-luciferase assay (g). The expression levels of CRYAB and KLF4 were detected by western blot (h).

Since CRYAB was found to be regulated by KLF4, we first inspected the genomic sequence upstream of CRYAB coding region by using the JASPAR software. Two putative KLF4 binding sites located 585-564 and 479-463bp upstream of CRYAB translational start site were identified (Figure 5a). To determine whether KLF4 binds the putative sites *in vivo*, we performed chromatin immunoprecipitation (ChIP) to detect the relevant DNA-protein interaction. As shown in Figure 2b, the CRYAB promoter in the nt - 479 to -463 region was detected in the KLF4 overexpressed MG63 cells. Similarly, the subsequent chromatin immunoprecipitation assay using anti-KLF4 antibody showed that the nt - 479 to -463 region was specifically present in anti-KLF4 immunoprecipitates, which was, however, diminished by KLF4 knockdown (Figure 5c). To further veritify the nt - 479 to -463 region was indeed responsive to KLF4, a series of pGL3-based luciferase reporter plasmids containing the wild type KLF4 binding region, a mutant binding region were generated (Figure 5d). These plasmids were individually transfected into MG63 cells with or without KLF4 overexpression, followed by the measurement by the transcriptional activities using luciferase assay. As shown in Figure 5e, pGL3 luciferase reporter plasmid containing the wild type KLF4 binding region, but not the mutant plasmid, showed a KLF4 responsive transcriptional activity in KLF4 overexpression cells (Figure 5e). Otherwise, comparing with the control cells, CRYAB promoter activity was substantially decreased in KLF4 stably knockdown cells (Figure 5f). Subsequently, to further prove that KLF4 regulated CRYAB expression dependent on its transcriptional activity, These plasmids containing wild type KLF4 or KLF4 ▵DBD or KLF4 ▵TA were individually transfected into the U2OS cells together with the pGL3-CRYAB WT, followed by the measurement by the transcriptional activities using luciferase assay. As shown in Figure 5g, the wild type KLF4, but not others, showed the increased transcriptional activity and the expression level of CRYAB was increased by the wild type KLF4 (Figure 5h).

### KLF4 promotes human osteosarcoma cell proliferation and migration via regulation of CRYAB expression

**Figure 6:**
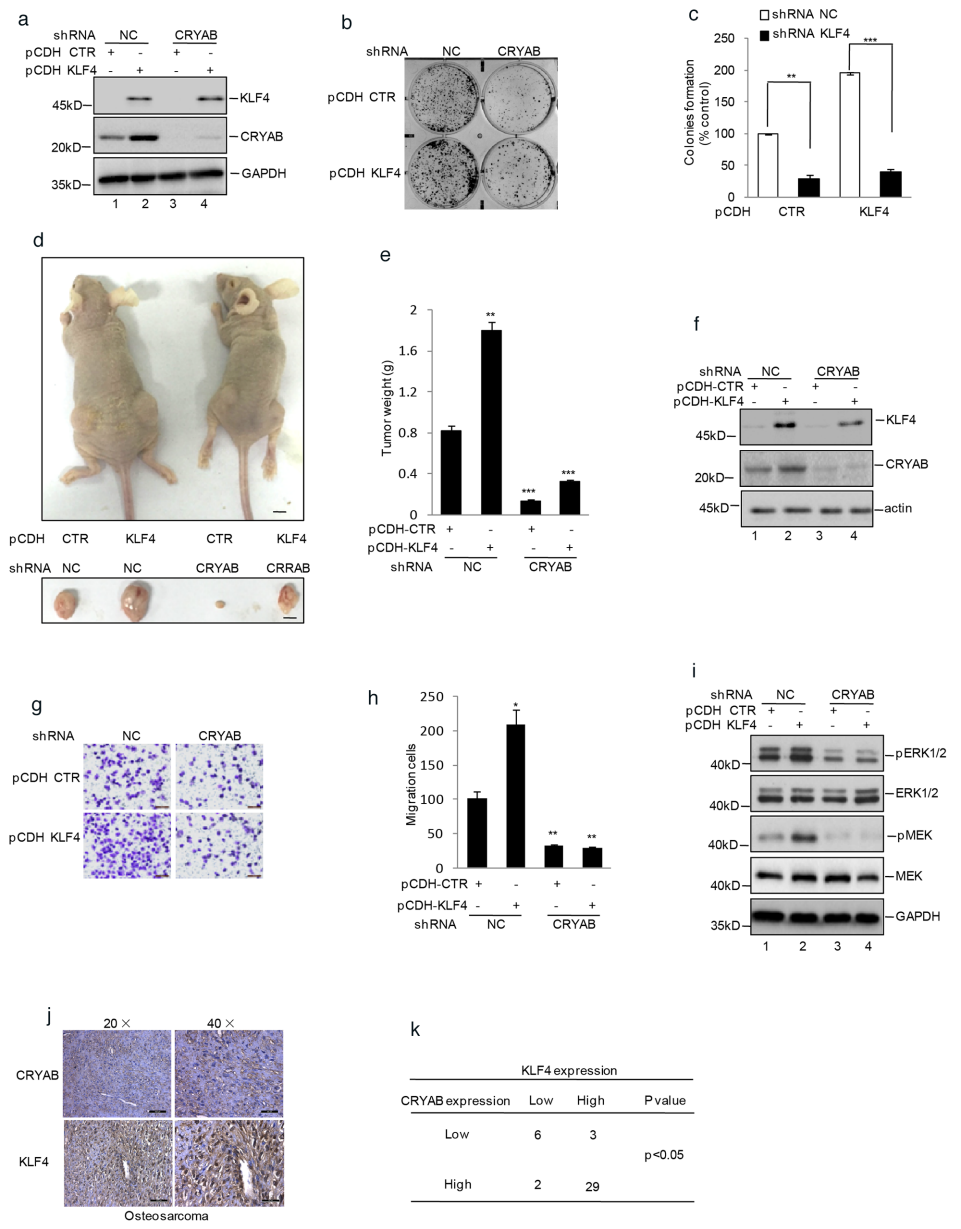
KLF4 promoted human osteosarcoma cell growth and migration by regulating CRYAB expression shRNA mediated knockdown CRYAB vector was transfected into the MG63 cells with or without overexpression of KLF4. **a–c.** Colony formation assay was used to measure the clonogenicity. **d–f.** The cells were injected subcutaneously into either side of the posterior flank of the same nude mouse (n =6 per group). The tumor size is shown in Figure 6d. The black bar represents 1 cm. The final tumor weight is shown in Figure 6e. The levels of KLF4 and CRYAB were detected by western blot (f). *P<0.05, **p<0.01 and ***p<0.001 vs Ctr. **g–h.** Matrigel migration assay was used to determine the migration viability of the cells. Migrated cells were plotted as the average number of cells per field of view. Results are representative of three independent experiments. *P<0.05, **p<0.01 and ***p<0.001 vs Ctr. **i.** ERK, MEK and their phosphorylation were detected by western blot assay. **j.** The immunostaining analysis of KLF4 and CRYAB protein expression from human osteosarcoma tissue microarray. High expression of KLF4 and CRYAB were shown by being stained as brown. **k.** The correlation between the expression of KLF4 and CRYAB in human osteosarcoma tissues from 40 patients was shown.

Recent studies have revealed that CRYAB enhanced osteosarcoma cells growth and metastasis [22]. Given that KLF4 played an important role in osteosarcoma tumorigenesis, we speculated that it might associate with upregulting CRYAB expression. To examine this, we overexpressed KLF4 using lentivirus vector in MG63 cells with or without stable knockdown CRYAB. As shown in Figure 6b and 6c, compared with the control cells, over-expressing KLF4 exhibit enhanced cell growth and migration. However, when CRYAB was knockdown, such stimulation effect of KLF4 was abolished, although effective expression of KLF4 was evident (Figure 6a and 6g–6h). To confirm it, we then adopted another methodology to investigate the effect of KLF4 and CRYAB on osteosarcoma tumorigenesis. Similarly, overexpression of KLF4 increased tumor volume and weight in the control cells but not in the CRYAB stable knockdown cells (Figure 6d–6f). CRYAB has been reported to activate the MAPK pathway. We detected the MAPK family protein expression, and verified the role of KLF4 in activating MAPK proteins via increasing CRYAB expression. As shown in Figure 6i, the phosphorylation of ERK and MEK was increased by KLF4 overexpression. Nevertheless, when CRYAB was decreased, the effect of KLF4 was abolished. After that, we analyzed the correlation between KLF4 and CRYAB in human osteosarcoma tissues and found that among 40 patient tumor tissue tested, 29 patients showed high expression of both KLF4 and CRYAB (Figure 6j–6k).

## DISCUSSION

In this study, we identified that the expression of KLF4 was markedly increased in osteosarcoma tissues compared with the normal bone tissues. The high levels of KLF4 were involved in human osteosarcoma cell proliferation, tumorigenesis, and migration. Afterward, mechanistic studies showed KLF4 specifically bound the promoter of CRYAB and transcriptionally regulated CRYAB expression in human osteosarcoma cells. In additional, we found that KLF4 enhanced osteosarcoma cell proliferation and migration through elevating CRYAB expression.

KLF4 was originally identified as a tumor suppressor in several cancers including gastrointestinal, esophageal, lung and pancreatic cancers [10, 16, 25, 26]. In breast cancer and squamous cell carcinoma, KLF4 was found to be an oncogenic protein [8, 28]. However, the role of KLF4 in osteosarcoma was not to be reported. In cultured and nude mouse xenografts of osteosarcoma cells, shRNA-mediated decreasing KLF4 expression inhibited cell proliferation, colony formation, and cell migration *in vitro*, and inhibited tumor growth, tumorigenesis *in vivo*. Conversely, KLF4 over-expression facilitated cell proliferation, colony formation, and cell migration *in vitro*, and accelerated tumor growth *in vivo*. In addition, the clinical analysis showed that the expression level of KLF4 was increased in human osteosarcoma tissues compared with the normal bone tissues. But the reason that KLF4 was increased in human osteosarcoma tissues was still not known. Therefore, it still need to be clarified in the future.

Previous studies have shown that KLF4 is an important zinc-finger transcription factor which transcriptionally regulates many genes [12]. Hence, to investigate the molecular mechanism, we screened the KLF4 regulated genes in osteosarcoma using mRNA array and found that CRYAB was a candidate gene. Furthermore, we found KLF4 upregulated CRYAB expression and bound the promoter of CRYAB. CRYAB, a human small heat-shock protein, has been found involved in a variety of processes including cytoskeletal assembly, remodeling and apoptosis inhibition [14]. CRYAB has been reported as a prognostic marker in several cancers, such as breast, renal, thyroid, nasopharyngeal, hepatocellular and lung cancers [7, 13]. Recent study has shown that the expression level of CRYAB was increased in osteosarcoma tissues and elevated CRYAB promotes osteosarcoma cell metastasis. Consistent with it, we also indicated CRYAB enhanced osteosarcoma cell migration and tumorigenesis and found that KLF4 promoted osteosarcoma cell migration and growth via CRYAB.

Taken together, our results indicate that the high level of KLF4 is a new adverse outcomes marker for OS patients and may be used as a new therapeutic target.

## MATERIALS AND METHODS

### Cell culture and reagents

Human osteosarcoma cancer cell line MG63, SaOS2 and U2OS were obtained from the American Type Culture Collection (ATCC). MG63 cells were cultured with MEM medium containing 10% fetal bovine serum (FBS; Gibco-BRL) and SaOS2 cells were cultured with L15 medium containing 10%fetal bovine serum (FBS; Gibco-BRL). U2OS cells were cultured with DMEM medium containing 10% fetal bovine serum (FBS; Gibco-BRL) Medium was renewed every one day and cells were passages before reaching confluence. The following antibodies were used in this study: antibody against CRYAB(Cell Signaling, USA; 8851); GAPDH (Santa Cruz Biotechnology, Dallas, TX, USA; SC-25778); KLF4 (Santa Cruz Biotechnology, Dallas, TX, USA; SC-126); KLF4 (Protein Tech, China, 11880-1-AP).

### Tissue microarrays and immunohistochemistry

Osteosarcoma tissue microarrays were purchased from Xi'anAlenabio(Xi'an, China) and it contained 40 osteosarcoma tissues and 20 normal bone tissues. The characteristics of the patients and their tumors were collected though review of medical records and pathologic reports. All patients had negative histories of exposure to either chemotherapy or radiotherapy before surgery, and there was no co-occurrence of other diagnosed cancers.

Tissue samples were processed according to routine procedures. In brief, the paraffin embedded osteoscarma tissue samples and the normal bone tissue samples were cut at 4 μm and mounted on glass slides. Then, the slides were deparaffinized, hydrated, and incubated in 3% H2O2 and microwaved to blockendogenous peroxidase activity. After 20 minutes to expose antigen hidden inside the tissue due to formalin fixation at room temperature, to inhibit non-specific antigen–antibody reactions possible in immunohistochemical staining, protein blocker was used for 5 minutes and the slides were washed thoroughly with PBS buffer. Then the slides were incubated overnight with the primary antibodies against KLF4 (1:200, rabbit polyclonal antibody) at 4 centigrade. Biotinylated goat anti-rabbit secondary antibody (1:200) was applied for 20 minutes at room temperature, followed by further washing with buffer to remove unbound antibody. A complex of avidin with horseradish peroxidase was then applied for 20 minutes at room temperature. For color development, the slides were stained with 3,3′diaminobenzidine, then were counterstained with hematoxylin.

The immunostaining analysis of KLF4 protein expression was assessed based on these tissue microarrays. The extent of the staining was used as criteria of evaluation. For each tissue sample, protein expression was scored according to the staining color: negative staining (no yellow), low staining (light yellow), moderate or high staining (yellowish brown or brown).

### Cell viability assay and colony formation assay

Cell viability was detected by CCK8 assay. Cells were plated in 24-well plates at a density of 5,000 cells in 200 μ1 medium per well 24 h before the experiment. Cell viability was examined by CCK8 assay. For colony formation assay, MG63 and SaOS2 cells were trypsinized and 1,000 or 750 viable cells were subcultured in 6-well plates (in triplicate). Cells were allowed to adhere and colonize for 14 days. To visualize colonies, media was removed and cells were fixed in 96% ethanol for 10 min and stained with crystal violet staining solution.

### Transfection and viral infection

Transient transfections were performed with Lipofectamine™3000 transfection reagent (Invitrogen) following the manufacturer's instructions. The shRNA sequences targeting KLF4 were5-TCCATTACCAAGAGCTCAT-3, and5-TGGACGGCTGT GGATGGAAA-3 and the shRNA sequence targeting CRYAB was 5-CCATTACTTCATCCCTGTCAT-3The shRNA was cloned using the PLKO.1 vector. Stable knockdown cells were established as previously described [4, 5].

Human KLF4 cDNA was inserted into the pCDH vector using the primers: F:5-GCGAATTC ATGAGGCAGCCACCTGGCGA-3 and R: 5-GCGGA TCC TTAAAAATGCCTCTTCATGT-3. KLF4cDNA was then amplified by RT-PCR using total RNA from MG63 cells. To generate lentivirus expressing KLF4, HEK 293T cells grown on a 6 cm dish were transfected with 2 ug pCDH-Flag-KLF4 or control vector, 1.5 ug psPax2, and 0.5 ug pMD2G. 24 h after the transfection, cells were cultured with DMEM containing 10% FBS for an additional 24 h. The culture medium containing lentiviral particles was centrifuged at 1,000 g for 5 min. Viral particles collected in the supernatant were used for infection. In order to establish the stable cell line, the puromycin was used as a selection marker for the infected cells. The expression efficiency was evaluated by western blot analysis [27].

### Real-time RT-PCR

Total RNA was isolated using Trizol (Invitrogen). One microgram of total RNA was used to synthesize cDNA using PrimeScriptTM RT reagent kit (Takara, RR047A) according to the manufacturer's instruction. The primers were used as following: KLF4 F:5-ACCTACACAAAGAGTTCCCATC-3; R 5- TGTGTTTACGGTAGTGCCTG-3. CRYAB:F:5-TGTTGGGAGATGTGATTGAGG-3 and R:5-GGG ATGAA GTAATGGTGAGAGGA-3. Actin:F:5-GACCTG ACTGAC TACCTC

ATGAAGAT-3and R:5-GTCACACTTCATGATGG AGTTGAAGG-3;

### Dual-luciferase reporter assay

MG63 cells were transfected withthe indicated luciferase reporter plasmid, together with or without KLF4. MG63 cells with or without knockdown KLF4 were tranfectedwith the indicated luciferase reporter plasmid. Renilla plasmid was contained in each transfection to normalize the transfection efficiency. The luciferase activities were measured by Dual-luciferase Reporter Assay system.

### ChIP assay

MG63 cells with overexpressed or knockdown KLF4 were crosslinked with 1% formaldehyde for 10 min at room temperature. ChIP assay was finished according to the manufacturer's instructions by using anti-Flag or anti-KLF4 antibody. The ChIP assay kit (Millipore, Merck KGaA, Darmstadt, Germany). Anti rabbitIgG were used as controls. The bound DNA fragments were elutedand amplified by PCR. The products were separated on 2% agarose gel by gel electrophoresis.

### Cell migration assay

MG63 cells were seeded in six-well plates. After 24h, cells were serum starved for 12h. A linear wound was created using a pipette tip and the cells were washed three times using PBS. After that, the cells were cultured in medium with 1% FBS. Wounds were then obtained after 24h. Three random images were taken at the time of the scratch and after 24h. The migration distance (units) was determined as reduction in the wound's gap using NIH Image-J software (National Institute of Health, Bethesda MD, USA).

MG63 cells were seeded in serum-free medium in chambers (8.00 mm pores, BD, Biosciences). Cells were allowed to migrate across inserts using serum-containing medium for 24h. The cells on the apical surface of the insert were scraped off and membranes with migrated cells were fixed in 1% paraformaldehyde and stained with crystal violet. Cell counts are expressed as the average number of cells per field of view. Three independent experiments were performed.

### Tumorigenicity assay in nude mice

Balb/c athymic nude mice were obtained from the Vital River Animal Laboratories (Beijing, China). All experiments involving animals were undertaken in accordance with the National Institute of Health Guide for the Care and Use of Laboratory Animals and with the approval of the Scientific Investigation Board of the General Hospital of PLA. MG63 cells as indicated were suspended in 0.1ml phosphate-buffered saline and then injected subcutaneously into the side of the posterior flank of the same female BALB/c athymic nude mice at 4 weeks of age. Tumor growth was measured daily using calipers, and the tumor volume was calculated according to the formula volume 1/4 length×width^2^×0.5.

### Statistics and data analyses

Data are expressed as the mean± SEM, and statistical evaluation was performed using one-way analysis of variance (ANOVA). Values of P<0.05 were considered as statistically significance.
